# Concomitant Dysregulation of Cerebral Vasoreactivity and Arterial Blood Pressure Is Closely Related in Patients with Carotid Stenosis

**DOI:** 10.3390/life16030472

**Published:** 2026-03-13

**Authors:** Hanga Pál, Rita Magyar-Stang, Borbála Csányi, Anna Gaál, Zsuzsanna Mihály, Zsófia Czinege, Péter Sótonyi, Tamás Horváth, Balázs Dobi, Dániel Bereczki, Akos Koller, Róbert Debreczeni

**Affiliations:** 1 Department of Neurology, Semmelweis University, 1083 Budapest, Hungary; pal.hanga@semmelweis.hu (H.P.); stang.rita@semmelweis.hu (R.M.-S.); gaal.anna@semmelweis.hu (A.G.); bereczki.daniel@semmelweis.hu (D.B.); 2 János Szentágothai Neurosciences School of PhD Studies, Semmelweis University, 1083 Budapest, Hungary; csanyiborbala10@gmail.com; 3 Department of Vascular and Endovascular Surgery, Semmelweis University, 1083 Budapest, Hungary; mihaly.zsuzsanna@semmelweis.hu (Z.M.); czinege.zsofia@gmail.com (Z.C.); sotonyi@hotmail.com (P.S.); 4 Research Center for Sport Physiology, Hungarian University of Sports Science, 1123 Budapest, Hungary; horvath2.tamas@tf.hu (T.H.); akos.koller@gmail.com (A.K.); 5 HUN-REN Neuroepidemiological Research Group, 1054 Budapest, Hungary; 6 HUN-REN-SE Cerebrovascular and Neurocognitive Disorders Research Group, Institute of Clinical Pathophysiology, Faculty of Medicine, Semmelweis University, 1083 Budapest, Hungary; 7 Department of Cell and Molecular Physiology, New York Medical College, Valhalla, NY 10595, USA

**Keywords:** cardiovascular autonomic nervous system, transcranial Doppler sonography, atherosclerosis, carotid artery disease

## Abstract

**Background:** In patients with severe atherosclerotic internal carotid artery stenosis (ICAS), the capacity of cerebral vasoreactivity (CVR)—an independent risk factor for cerebral ischemia—is reduced, and dysregulation of arterial blood pressure (ABP) may also be present. Thus, this study assessed the relationship between changes in cerebral blood flow velocity (BFV) in response to vasoactive stimuli (as measured by transcranial Doppler (TCD)), characterizing CVR and cardiovascular autonomic nervous system (CANS) function. **Methods:** Common carotid artery compression (CCC n = 26), hyperventilation (HV) and breath-holding (BH) tests (n = 31), and the Valsalva maneuver (VM n = 34) were used to assess CVR in patients with ICAS. In the middle cerebral arteries, BFV was monitored by TCD, whereas ABP was registered non-invasively. For statistical analysis, validated indices describing CANS function—namely, sympathetic index (SI), pressure recovery time (PRT), and Valsalva heart rate ratio (VHRR)—were selected based on the VM response. Several parameters were defined in order to evaluate CVR responses, including cerebral arterial resistance (CAR = ABP/BFV), which was correlated with the CVR indices using Spearman’s pairwise correlation and canonical correlation. **Results**: A significant correlation was found between several CVR indices of the HV-BH and VM tests and CANS indices of VM using Spearman’s pairwise correlation test. Regarding the HV-BH CVR and CANS indices of VM, a significant correlation was found between CAR values until it reached its maximum on the to-be-operated side (CAR_timetomaxICAop_) and VHRR (*p* = 0.041). A significant correlation was also found between the time elapsed until the CAR minimum value (CAR_timetominICAop_) and SI (*p* = 0.019). Concerning the CVR and CANS indices of the VM, a significant correlation was found between cerebrovascular Valsalva ratio on the to-be-operated side (CVAR_ICAop_) and PRT (*p* = 0.002). Canonical correlation analysis confirmed that impairments of CANS and CVR may be associated. **Conclusions**: In patients with severe ICAS, the potentially concomitant dysregulation of cerebrovascular reactivity and the cardiovascular autonomic nervous system can further increase cerebral ischemic risk.

## 1. Introduction

Cerebrovascular reactivity (CVR) represents the capacity of the cerebral small vessels to adjust their diameter in response to arterial blood pressure oscillations and the metabolic demands of the brain [1]. Severe internal carotid artery stenosis (ICAS) in atherosclerosis can lead to the impairment of CVR, as has been shown in several previous studies using functional transcranial Doppler (TCD) [2,3,4]. In humans, TCD is suitable for measuring the following changes in blood flow velocity (BFV) of large intracranial cerebral vessels, such as the middle cerebral artery (MCA) [5,6]. Several validated stimuli can be used to estimate CVR based on BFV changes measured by TCD. One example is the common carotid artery compression (CCC) test, in which temporary cerebral hypoperfusion is followed by a transient reactive hyperemic response (THR) [7,8,9]. Another is CO_2_ reactivity, which can be evoked by modifying blood CO_2_ level (5% CO_2_ inhalation, hyperventilation (HV), and breath-holding (BH)) [10,11,12,13]. Another stimulus is the so-called Valsalva maneuver (VM), which elicits simultaneous changes in perfusion pressure and thus blood flow in the cerebrovascular system, evoking changes in cerebrovascular resistance [14,15,16,17,18,19,20]. The significant changes in cardiac output and the resulting changes in arterial blood pressure produced during the Valsalva maneuver, in addition to eliciting cerebral vascular reactions, also stimulate arterial baroreceptors, resulting in characteristic changes in heart rate (HR) and arterial blood pressure (ABP) [21]. The dynamics and extent of these reactions can be used to characterize the function of the cardiovascular autonomic nervous system (CANS). Thus, VM allows for assessing both CVR and CANS function at the same time [22,23].

Central control of the arterial blood pressure is ensured by the baroreflex mechanism, which maintains the range of ABP spontaneous changes within a relatively narrow range [24,25,26]. Integrity of the baroreflex can be impaired by changes in the structure of the vascular wall (stiffness) due to many factors, such as age, diabetes, hypertension, and atherosclerosis [27,28,29,30,31,32,33].

We have previously shown that changes occur in BFV in the MCA elicited by functional TCD tests in patients with severe ICAS [3,34,35]. Several CVR parameters have been shown to be sensitive to hemodynamic disturbances. Interestingly, we observed that most patients also presented signs of cardiovascular autonomic dysfunction [35].

Based on previous results [36], we hypothesized that in patients with severe ICAS, there may be a correlation between cerebrovascular reactivity and the severity of cardiovascular autonomic dysregulation. This correlation may help identify patients with significant carotid artery stenosis, who tend to have an increased risk of cerebral ischemic events.

## 2. Materials and Methods

The basic characteristics of the present study are identical to our previous one: a single-center exploratory study (approved by the Semmelweis University Regional and Institutional Committee of Science and Research Ethics (SERKEB, Number: 256/2018)) and registered at ClinicalTrials.gov (Identifier: NCT03840265). The TCD examinations were performed in the Doppler Laboratory of the Department of Neurology, Semmelweis University.

The study was carried out in accordance with the principles outlined in the Declaration of Helsinki. Patients admitted to the Department of Vascular Surgery of Semmelweis University for carotid endarterectomy (CEA) due to significant ICAS were consecutively enrolled between 15 January 2019 and 30 September 2021 after providing written consent. The severity of ICAS was established by computed tomography angiography in accordance with the North American Symptomatic Carotid Endarterectomy Trial (NASCET) criteria, and a stenosis of ≥70% was defined as hemodynamically significant. Patients were under continuous clinical monitoring throughout the investigations.

### 2.1. Patients’ Baseline Characteristics

A total of 48 eligible patients had bilateral TCD registrations (male: 36, mean age ± SD: 68.08 ± 7.23 years; female: 12, mean age ± SD: 70.16 ± 7.35 years). Since not all the tests were technically appropriate, the following tests were evaluable: CCC test for 26 patients, HV and BH tests for 31 patients, and VM test for 34 patients. Among the patients who underwent HV-BH and VM tests, six had significant bilateral ICAS, as did four of the patients who underwent CCC. The patients were asymptomatic with respect to ICAS and were treated with antiplatelet therapy in addition to antihypertensives and statin medication. In the remaining patients, the contralateral stenosis did not exceed 70%. The purpose of bilateral TCD monitoring was to measure changes in the BFV in the MCA on the side of severe ICAS and on the contralateral side and to compare the differences. The two sides were defined as follows: the side to be operated on as ICA_op_ and the contralateral side not to be operated on as ICA_nonop_. TCD measurements were performed before CEA. There were no adverse events during the functional TCD maneuvers. Table 1 summarizes patients’ data.

### 2.2. TCD Measurements and Protocols

Bilateral middle cerebral artery (MCA) blood flow velocity (BFV, cm/s) was measured using 2 MHz transcranial Doppler (TCD) probes (DWL Multi-Dop T2, Compumedics Germany GmbH, Sipplingen, Baden, Germany) with participants in a semi-sitting position via the transtemporal window at a depth of 45–55 mm. During TCD measurements, continuous, non-invasive, beat-to-beat ABP was measured by radial artery applanation tonometry (Colin-BP508, Colin Medical Technology Corporation, Hayashi, Komaki, Aichi, Japan). Heart rate (HR) was continuously measured with an electrocardiogram (ECG).

To compute beat-to-beat MBFV and MABP, we used Equations (1) and (2):

MBFV = (BFV_sys_ + 2 BFV_dia_)/3 (1)

MABP = (ABP_sys_ + 2 ABP_dia_)/3 (2)

where BFV_sys_ is the maximal systolic, BFV_dia_ is the end diastolic velocity, ABP_sys_ is the peak systolic, and ABP_dia_ is the end diastolic arterial blood pressure.

The CCC test, CO_2_ reactivity by hyperventilation (HV) and breath-holding (BH) test, and VM test were used as previously described, and several indices were calculated [3,34,35]. Several of them were sensitive to hemodynamic disturbances in our previous publication [35].

### 2.3. Data Processing

Analog signals from four channels (TCD1, TCD2, ABP tonometry, and ECG) were digitized simultaneously at a sampling rate of 500 Hz. The raw data were saved in European Data Format files, which were then imported, digitally filtered, and segmented by using LabChart software (ADInstruments, LabChart v8, Colorado Springs, CO, USA). Preprocessed segments were exported as separate text files for further analysis. Custom Python scripts (Python v3.12.2) were used to process these files, performing linear interpolation of the cardiac cycle data into 0.5 s equidistant intervals. The interpolated data were exported to Microsoft Excel for Microsoft 365 (Version 2602).

### 2.4. Common Carotid Artery Compression Test (CCC Test)

**Figure 1:**
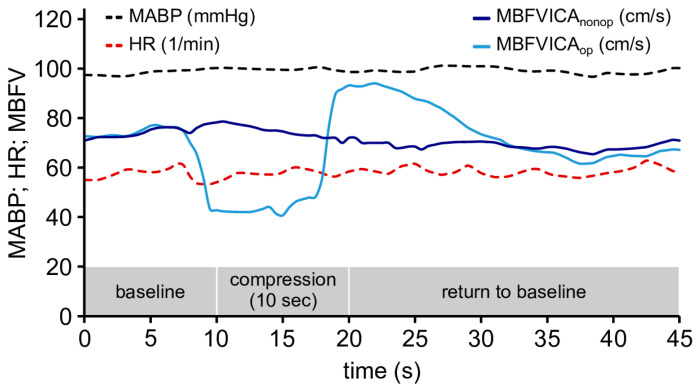
Original recordings of the common carotid artery compression (CCC) test of a patient with significant internal carotid artery stenosis (ICAS). Changes in mean blood flow velocity (MBFV), mean arterial blood pressure (MABP), and heart rate (HR) are shown during 10 s of manual compression of the common carotid artery (CCA). During compression of the carotid artery, MBFV in the ipsilateral MCA (MBFV_ICAop_) decreased significantly. After the release of the compression, a transient hyperemic response (THR) occurred. MABP and HR did not change during the CCC test.

The CCC test was performed after a carotid duplex ultrasound (CDS) examination. Prior to the examination, high-risk, rupture-prone plaques were screened out using CDS to ensure the examination was safe. The variables were defined according to our previous publication, including transient hyperemic response ratio (THRR) and return to baseline (RTB) [35]. Figure 1 illustrates the procedure of the CCC test.

The indices of the BFV changes during the CCC tests are as follows:

(1) Return to baseline (RTB): The interval between the release of carotid compression and the return of MCA velocity values to baseline was recorded.
(2) Transient hyperemic response ratio (THRR):

$$\mathrm{THRR}=\frac{\left(F3-F1\right)}{F1}$$

whereas F1 reflects the baseline BFV, and F3 reflects the BFV value after the release of the compression. THRR was calculated from the peak and mean systolic velocity values (PSV and MBFV) [8].

### 2.5. Hyperventilation (HV) and Breath-Holding (BH) Test

**Figure 2:**
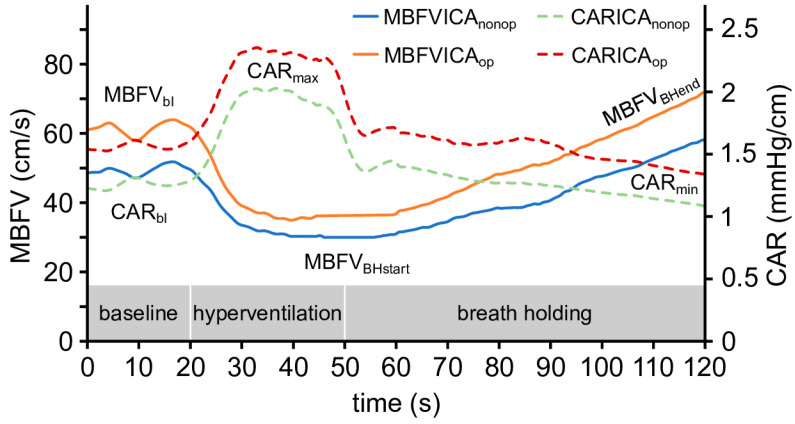
Original recordings of the combined hyperventilation and breath-holding maneuver of a patient with ICAS. Changes in mean blood flow velocity (MBFV) in the middle cerebral artery (MCA) and cerebral arterial resistance (CAR) during the hyperventilation and breath-holding tests are presented. Specifically, 30 s of HV induced a substantial decrease in MBFV, likely due to cerebral vasoconstriction elicited by reduced PaCO_2_. Hyperventilation was followed by breath-holding, with a duration that varied across individuals. During breath-holding, the MBFV increased, likely due to cerebral vasodilation elicited by the increase in PaCO_2_. CAR change during the tests was also calculated by the following formula: CAR = MABP/MBFV. The changes in CAR showed an opposite trend to the changes in MBFV.

The total CO_2_ reactivity of cerebral vessels was assessed by HV and BH maneuvers. The patients hyperventilated for thirty seconds and were then instructed to hold their breath for as long as possible. Several time and velocity variables were defined. Furthermore, changes in cerebral arterial resistance (CAR) were calculated by the ratio of mean arterial blood pressure (MABP) and mean blood flow velocity (MBFV), expressed as CAR = MABP/MBFV, as previously described [35]. In Figure 2, changes in MBFV and CAR elicited by HV and BH are illustrated.

Definitions of the indices obtained during the HV-BH tests are as follows:

(1) Hyperventilation index (HVI): HVI was calculated according to the following formula:

$$\mathrm{HVI}=\frac{\Delta MBFVHV}{MBFVbl}\times 1/30\times 100$$

(2) Time to the minimum value of mean blood flow velocity during hyperventilation (time to MBFV_HVmin_): The time elapsed from the beginning of HV until MCA MBFVHVmin.
(3) Breath-holding index (BHI): BHI was calculated according to the following formula [13,37]:

$$\mathrm{BHI}=\frac{\left(MBFVBHend-MBFVBHstart\right)}{MBFVBHstart}\times 100\times \frac{1}{tBH}$$

(4) Time from the baseline CAR values until the maximal CAR values (CAR_timetomax_): The time elapsed from CAR_bl_ until CAR_maxHV_.
(5) Time from the maximum CAR value until the minimal CAR value (CAR_timetomin_): The time elapsed from CAR_max_ to CAR_min_.

### 2.6. Valsalva Maneuver

VM elicits considerable changes in ABP and BFV in cerebral vessels such as the MCA. The responses to VM can be divided into four stages, which are suitable for assessing both CVR and the function of CANS [14]:

- Stage I: The rise in intrathoracic pressure is transmitted to the aorta, leading to a transient increase in ABP.
- Stage IIa (early): The elevated intrathoracic pressure reduces venous return to the heart, resulting in a decreased stroke volume and a progressive decline in ABP.
- Stage IIb (late): Reduction in ABP is sensed by baroreceptors in the carotid arteries, activating the sympathetic cardiovascular autonomic nervous system. This response increases peripheral vascular resistance and heart rate, leading to a subsequent rise in ABP.
- Stage III: After ceasing forced expiration, a sudden drop in the intrathoracic pressure occurs, causing a temporary decrease in ABP.
- Stage IV: An overshoot (OS) in ABP is observed due to the previously elevated peripheral vascular resistance and the increased venous return.
- VMs were performed in a standardized manner (same position and duration, creating an intrathoracic pressure of 40 mmHg, repeated three times).

In cases of cardiovascular autonomic dysfunction, impaired responses in BP/HR can be observed, as indicated by the lack of a compensatory increase in ABP in phases IIb and IV.

The cardiovascular autonomic nervous system indices of VM are as follows:

Several indices have been validated for characterizing the cardiovascular autonomic function, including the sympathetic index (SI), the Valsalva heart rate ratio (VHRR), and the pressure recovery time (PRT) [15,18,19,38].

**Sympathetic Index (SI):** Represents the percentage change in MABP measured at the end of phase II (MABP_IIend_) relative to the baseline MABP (MABP_bl_). A normal SI value is ≥0 [18]. Patients with CANS dysfunction exhibit a negative SI value. SI is calculated as

SI = (MABP IIend − MABP BL)/MABP BL × 100

**Valsalva Heart Rate Ratio (VHRR):** Defined as the ratio of the maximum heart rate (HR_max_) during the Valsalva maneuver to the minimum heart rate (HR_min_) recorded within 30 s after the maximum heart rate (HR_max_) [15,19]. In individuals over 60 years of age, a normal VHRR is >1.35 [15]. Values below 1.35 indicate cardiovascular autonomic dysfunction. It is calculated as

VHRR = HR_max_/HR_min_

**Pressure Recovery Time (PRT): Refers to the time interval (in** seconds) between the lowest systolic ABP in stage 3 (t1) and the point at which systolic ABP returns to the BL values in stage 4 (t2). A normal PRT is less than 4 s [15,38]. Patients with a PRT exceeding 4 s are considered to have cardiovascular autonomic nervous system dysfunction. PRT is calculated as follows:

PRT = t2 − t1

**Figure 3:**
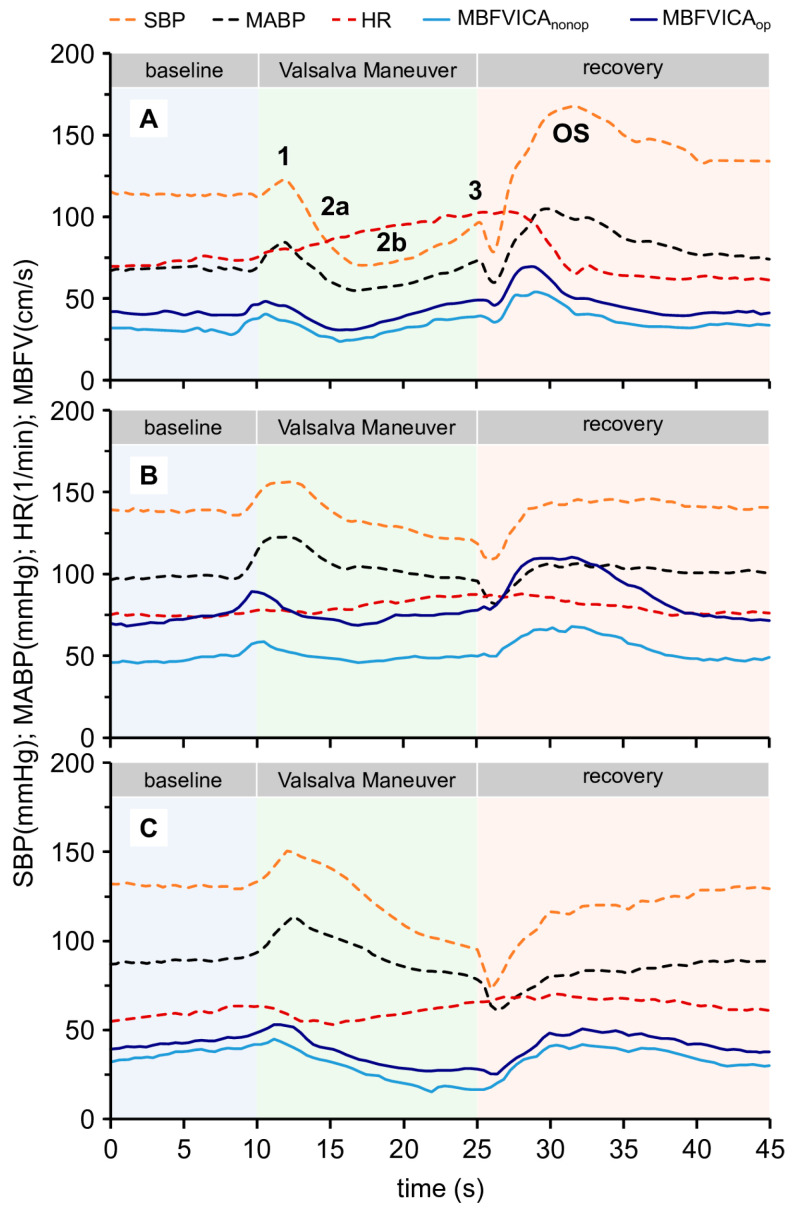
Original recordings of the Valsalva maneuver of three patients with significant internal carotid artery stenosis (ICAS). (**A**) The physiologic changes in the middle cerebral artery (MCA) mean blood flow velocity (MBFV), arterial blood pressure (ABP) (systolic and mean), and heart rate (HR) during VM. The first stage (1) is the beginning of the maneuver, where ABP and MBFV increase. In stage 2a, ABP and MBFV progressively decrease. In stage 2b, a compensatory increase in ABP and MBFV begins and continues until stage 3. Stage 3 marks the end of VM with a transient decrease in MABP and MBFV, which is followed by an overshoot (OS) reaction. Regarding heart rate (HR), a continuous increase can be observed until OS. (**B**,**C**) Two types of pathological responses. In (**B**), the increases in stages 2b and OS are minimal regarding the ABP curves. In (**C**), both ABP and MBFV progressively decrease in stages 2b and OS. The increase in HR is also absent.

Figure 3 shows changes in systolic arterial blood pressure (SBP), mean ABP (MABP), MCA mean BFV (MBFV), and heart rate (HR) during VM in normal and pathologic conditions. Similar to the other vasoactive stimuli, there are several indices describing VM.

The relevant parameters, which correlate with the dysfunction of CANS and CVR, are as follows:

(1) Time to stage IIb of the Valsalva maneuver (time to 2b): Temporal variables represent the duration until the onset of stage 2b.
(2) Time to stage overshoot of Valsalva maneuver (time to OS): Temporal variables represent the duration until the onset of stage OS.
(3) Centro-peripheral Valsalva ratio (CPVR): Represents the changes in the MFBV and MABP between stages 2b and 3 [39]:

$$\mathrm{CPVR}=\frac{MBFV3-MBFV2b}{MABP3-MABP2b}$$

(4) Centro-peripheral overshoot index (CPOI): Based on CPVR, CPOI is defined, representing the difference between the OS and BL values of the mean BFV in the numerator, while the denominator corresponds to the difference between the OS and BL values of the MABP.

$$\mathrm{CPOI}=\frac{MBFVos-MBFVbl}{MABPos-MABPbl}$$

(5) Cerebrovascular Valsalva ratio (CVAR): Represents the increase in the MBFV in stage 2b compared to stage 3 [39,40]:

$$\mathrm{CVAR}=\frac{MBFV3-MBFV2b}{t3-t2b}$$

### 2.7. Statistical Analysis

The main purpose of the study was to correlate CVR variables measured on the ICA_op_ side with autonomic variables of the VM to evaluate the relationship between CVR impairment and cardiovascular autonomic dysfunction in patients with ICAS. The parameters characterizing CVR, calculated based on the CCC, HV-BH, and VM tests, were correlated with the indices indicating cardiovascular autonomic function (SI, PRT, and VHRR). The availability of paired data allowed for correlation of the CVR indices from the CCC test, the HV-BH test, and the VM with the autonomic indices derived from the VM. The number of elements of the correlations (n) is shown in Table 2, Table 3 and Table 4.

Pairwise Spearman’s and canonical correlation analyses were used to quantify the relationship between variables. Canonical correlation analysis is a statistical method used for assessing and maximizing correlations between two sets of variables [41]. Only those CVR variables were kept for canonical correlation analysis that had pairwise correlations above or equal to 0.3 (in absolute value) with at least one autonomic index. In addition, CVR variables with <30 observations were excluded. The statistical significance of the canonical correlation coefficients was tested using the F-approximation (asymptotic) of the Pillai–Bartlett trace.

A significance threshold of *p* < 0.05 was applied for all tests. Statistical analyses were conducted using the TIBCO Statistica^®^ 13.5.0 program and the R programming language version 4.4.1. Data visualization was performed with Microsoft Excel software, InkScape 1.4 software, and the R programming language version 4.4.1.

## 3. Results

### 3.1. Pairwise Spearman’s Correlations

Using the established cut-off values for the cardiovascular autonomic indices from the VM, an abnormal SI was found in 23 patients, VHRR in 15 patients, and PRT in 8 patients. Several CVR indices of the three functional tests have been described previously [35]. The results of Spearman’s pairwise correlation analysis of the CVR indices, which showed significant correlation with the cardiovascular autonomic indices of VM, are summarized in Table 2, Table 3 and Table 4.

#### 3.1.1. Correlation Between Changes in CVR in Response to CCC Test and CANS Indices

The indices of the CCC test described previously were correlated with the CANS indices of the VM. There was no significant correlation observed with any of them.

#### 3.1.2. Correlation Between HV-BH Test CVR and CANS Indices

A positive, significant correlation was found between CAR_timetomaxICAop_ and VHRR (*p* = 0.041), while a positive, significant correlation was observed between CAR_timetominICAop_ and SI (*p* = 0.019).

#### 3.1.3. Correlation Between VM CVR and CANS Indices

A positive, significant correlation was found between CVAR_ICAop_ and PRT (*p* = 0.002).

### 3.2. Canonical Correlation Analysis

The following eight reactivity indices satisfied the pairwise correlation and sample size requirements: time to MBFV_BHblICAop_, BHI_ICAop_, CARmax_ICAop_, CAR_timetomaxICAop_, CAR_timetominICAop_, CAR_AUCHVICAop_, CPOI_ICAop_, and CVAR_ICAop_. In total, 17 patients were included in the canonical correlation analysis, as complete cases were required across all variables in both the CVR and autonomic domains. Table 5 lists the canonical correlation coefficients and related test results for all three canonical variates (in each set of variables—CVR or CANS indices). The first line shows the coefficient for the first canonical variate and the significance of all three canonical variates taken together; the second shows the coefficient for the second variate and the combined significance for the second and third variates; the last line shows the third variate alone.

The first canonical variate for the reactive set was mostly determined by CAR_timetominICAop_ and CAR_timetomaxICAop_. The second canonical variate was mostly determined by the same two indices in the opposite direction and by CAR_HVAUC-ICAop_. The first variate from the autonomic set was mostly determined by PRT and SI. For the second variate, SI was dominant. The canonical correlation coefficient was 0.782 between the first sets of autonomic and reactive variates. For the second canonical variate, the canonical correlation was somewhat lower at 0.725. While relatively strong correlations were observed for these (above 0.7), none were significant.

## 4. Discussion

By measuring blood flow velocity in the middle cerebral arteries, arterial blood pressure, and heart rate in response to various vasoactive stimuli, this study showed a significant correlation between changes in the indices of the cerebral vasoreactivity and autonomic indices of the Valsalva maneuver, suggesting that in many patients with significant internal carotid artery stenosis (ICAS), there is a simultaneous impairment of cerebral vasoreactivity and the cardiovascular autonomic system.

### 4.1. Interpretation of the Results

#### 4.1.1. Common Carotid Artery Compression Test

Correlation tests with the variables of cerebral vasoreactivity of the common carotid artery compression (CCC) test and the autonomic indices of the VM did not demonstrate significant correlation. This finding may be explained by the fact that the response to the CCC stimulus is significantly influenced by the number and capacity of the anastomoses of the circle of Willis. Thus, the cerebrovascular reactivity (CVR) indices derived from the CCC carry complex information, including the modifying effect of the available anastomoses of the circle of Willis, in addition to the regional carotid cerebral vasoreactivity.

#### 4.1.2. Hyperventilation, Breath-Holding Test, and Valsalva Maneuver

The HV-BH and VM tests affect the entire cerebral vasculature; thus, the anastomotic role of the circle of Willis is negligible. The significant relationship between HV-BH and VM CVR indices and CANS functions likely indicates valid mechanistic connections.

#### 4.1.3. Interpretation of the Canonical Correlation

The canonical correlation evaluates the strongest possible linear relationship between the CVR and CANS indices. Based on the canonical variates, the strongest relationship is observed between the CAR_timetomax_ variable of the CVR indices and the PRT and SI variables of the CANS indices. In other words, out of the many defined CVR variables of the three vasoactive tests, only a few are sensitive to the hemodynamic disturbance caused by ICAS. In addition, the autonomic indices of VM do not designate identical, matching patient groups, so neither index is likely to be suitable to adequately verify vasoreactivity disorder and associated CANS dysfunction. The current clinical significance of the canonical correlation results lies in the relatively strong relationship observed between the aforementioned indices. Therefore, the impairment of CVR and CANS in advanced atherosclerosis is not independent of each other in many cases, and both aspects have clinical significance in assessing the risk of cerebral ischemia.

### 4.2. Clinical Significance of Cardiovascular Autonomic Dysfunction Associated with Carotid Stenosis and Its Relationship to the Findings of the Present Study

The patient population and the method in the present study differ from those of previous studies that have already drawn attention to the possibility of atherosclerosis and the often associated cardiovascular autonomic dysfunction. In the present study, we assessed cardiovascular autonomic function based on the validated autonomic indices of the Valsalva maneuver, which is reproducible and suitable for the examination of cerebral vasoreactivity. The significant correlation of dysfunction-vasoreactivity disorder was also confirmed using this approach. We consider the VM to be a highly informative test that is easy to perform, standardized, and extremely valuable in everyday clinical practice; according to our experience, the technique is reproducible even in elderly patients.

**Figure 4:**
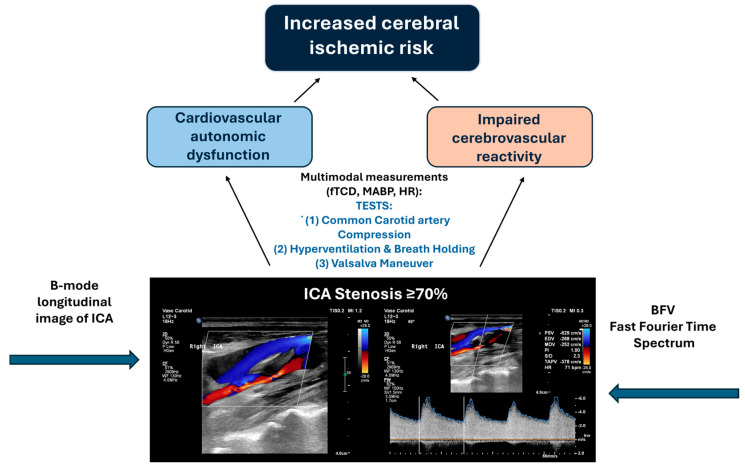
Chart flow of the present study. Assessment of the function of cerebrovascular reactivity and the cardiovascular autonomic nervous system in patients with severe internal carotid artery stenosis: three functional transcranial Doppler (TCD) tests were used to assess cerebrovascular reactivity, and the integrity of the cardiovascular autonomic nervous system was determined based on parameters of the validated autonomic indices of the Valsalva maneuver, namely sympathetic index, Valsalva heart rate ratio, and pressure recovery time. Both reduced cerebrovascular reactivity and cardiovascular autonomic dysfunction may represent serious cerebrovascular risk factors. The image depicting severe internal carotid artery stenosis was obtained using duplex Doppler ultrasound in our laboratory and Microsoft PowerPoint for Microsoft 365 (Version 2602) was additionally use to prepare this figure.

The idea of correlating these parameters arose from the fact that recognizing autonomic dysfunction in this patient population might be relevant, since it can also enhance their cerebral ischemic risk, among other clinical features (Figure 4) [42].

The autonomic dysfunction in patients with significant ICAS might have the following significant clinical relevance:

(1) Based on the results of previous studies, the concomitant impairment of CVR and CANS function should be expected in atherosclerotic patients with ICAS [36,43,44,45].
(2) Regarding procedural relevance during carotid endarterectomy (CAE), impaired cerebrovascular reactivity combined with cardiovascular instability may result in poor arterial blood pressure control and may enhance the risk of low cerebral perfusion [46,47].
(3) Cardiovascular autonomic nervous system dysfunction is associated with cerebral ischemia, carries prognostic significance, may worsen functional outcomes, and may increase cardiovascular mortality [48,49,50,51,52].

#### 4.2.1. The Significance of Multimodal Functional Methods, Like TCD with ABP Monitoring: Relationship Between Cardiovascular Autonomic Dysfunction and Cerebrovascular Reactivity

The monitoring of cerebral blood flow velocity, arterial blood pressure, and heart rate allows simultaneous assessment of cerebral and systemic circulatory changes and their interactions in response to vasoactive stimuli. Measuring BFV with the transcranial Doppler method is widely accepted for testing the effects of autonomic dysregulation on cerebral perfusion [43,53]. This method is applicable in the diagnosis of vasovagal syncope, orthostatic intolerance, chronic autonomic failure, and baroreflex failure. The estimation of the risk of cerebral ischemia in patients with carotid stenosis is of great importance, especially in the preoperative period. It has been proven that impaired cerebrovascular reactivity is associated with a higher risk of cerebral ischemia, cognitive decline, and worse clinical outcome in ischemic stroke [48,50].

#### 4.2.2. Cardiovascular Autonomic Nervous System Dysfunction as a Risk of Ischemic Stroke

Baroreceptors are an important part of beat-to-beat regulation of ABP via the arterial baroreflex, which maintains ABP within a relatively narrow range despite significant changes in body position or cardiac output [26]. In patients with ICAS of atherosclerotic origin, the vascular stiffening of the vessel wall can decrease the sensitivity of baroreceptors in the carotid bulb due to decreased distensibility, which can lead to reduced baroreflex sensitivity via the impaired afferent signaling of the glossopharyngeal and vagal nerves [24]. The reduced effectiveness of the baroreflex can result in significant oscillations of arterial blood pressure, which, under extreme circumstances (e.g., during surgery), may exceed the pressure limits of cerebral pressure/flow autoregulation, resulting in critical cerebral hyperperfusion or hypoperfusion [46,47,54]. The following phenomena are manifestations of impairment of CANS: decreased baroreflex sensitivity, decreased heart rate variability, sympathovagal imbalance, and increased arterial blood pressure variability [52,55]. Several previous studies have verified the relationship between the impairment of the autonomic nervous system and a higher risk of cerebral ischemic events and myocardial infarcts [50,51,56,57].

Based on these, we suggest that it is clinically relevant to consider the impairment of CANS in patients with significant carotid artery stenosis who are candidates for carotid reconstruction, together with a complex assessment of CVR impairment to assess cerebral ischemic risk.

The present study complements our previous publication, in which we evaluated several cerebrovascular reactivity variables, of which flow resistance was found to be sufficiently sensitive to hemodynamic disturbance caused by ICAS. Our current results showed that, in addition to the well-known breath-holding index, parameters mainly indicating changes in cerebrovascular resistance correlated with autonomic indicators of the Valsalva maneuver. This result further strengthens the previous finding that a more comprehensive preoperative examination may require monitoring of other vital parameters, such as arterial blood pressure and heart rate, to accurately assess the risk of cerebral ischemia.

#### 4.2.3. Periprocedural Complications

In the present study, another aspect was considered. In the patient population, cerebral blood flow velocity was measured by functional TCD immediately before carotid endarterectomy, which has clinical importance. During the surgical procedure, cerebral perfusion pressure may temporarily decrease due to carotid artery clamping. In the presence of severe hemodynamic instability and insufficient arterial blood pressure control, this may represent an additional significant risk of cerebral ischemia in this patient population. Considering all of this, the preoperative assessment of CANS function is important [46,58]. Furthermore, the dilatative and constrictive vasoreactivity capacity of cerebral arterioles may be significantly reduced by prolonged hypoperfusion caused by persistent carotid stenosis. Surgical reconstruction of carotid stenosis eliminates the risk of cerebral hypoperfusion, but the stabilization of vasoreactivity capacity does not occur immediately after the reconstruction procedure. In the case of prolonged stabilization of constrictive capacity, the risk of post-reconstruction hyperperfusion syndrome is clinically significant. This is especially true if, as a result of cardiovascular autonomic dysfunction, reduced sensitivity, or absence of the baroreflex (bilateral carotid stenosis), the oscillation of arterial blood pressure becomes so significant that it creates the basis for the development of focal cerebral hyperperfusion. These observations are supported by previous studies [59,60]. Due to the frequent concomitant dysfunctions of cerebral vasoreactivity and arterial blood pressure regulation—as demonstrated by the present findings—the development of postoperative focal hyperperfusion syndrome should be expected in this group of operated patients. Thus, prolonged postoperative monitoring of arterial blood pressure and MCA blood flow velocity should be an essential aspect for optimal patient care.

### 4.3. Strength of the Study

The strength of our study is that we were able to evaluate cardiovascular and cerebrovascular responses in a complex manner in response to the Valsalva maneuver and other vasoactive stimuli, which were easily and safely performed even in relatively elderly patients with significant carotid artery stenosis. From a neurological point of view, it is known that reduced cerebral vasoreactivity is an independent risk factor for cerebral ischemia. Based on our results, which are also in line with the findings of previous publications [36]—including the assessment of cardiovascular autonomic dysfunction as a risk factor for cerebral ischemic events, especially before CEA—it may be useful in preventing perioperative neurological complications caused by intraoperative clamping due to the depletion of cerebrovascular reserve capacity.

### 4.4. Limitations

A limitation of the present study is the relatively small number of patients included, which was due to the following reasons: (1) the absence of an adequate bilateral temporal acoustic window and (2) strict TCD protocol requirements combined with the selection criteria; only those registrations were evaluated in which the signal-to-noise ratio was excellent. Due to the low number of patients examined, it was not possible to include additional control variables enabling subgroup analyses.

## 5. Conclusions

The findings of the present study suggest that in patients with severe internal carotid artery stenosis, a significant correlation exists between the reduced capacity of cerebral resistance vessels and the impairment of the cardiovascular autonomic nervous system. One potential underlying mechanism is impaired baroreceptor signaling due to remodeling (stiffening) of large extracranial arteries, resulting in blood pressure instability, which leads to increased risk of cerebral hypoperfusion and ischemia, especially during endovascular surgical procedures.

## Figures and Tables

**Table 1:** Cardiovascular risk factor of the studied patients.

n	Mean Age ± SD:	Sex	Hypertension	Diabetes	Ischemic Heart Disease	Smoking	Hyperlipidaemia
n_(all)_ = 48	male: 68.08 ± 7.23 female: 70.16 ± 7.35	n_male_ = 36 n_female_ = 12	n = 44 (91.7%)	n = 44 (91.7%)	n = 14 (29.2%)	n = 16 (33.3%)	n = 41 (85.2%)

**Table 2:** Results of Spearman’s pairwise correlation test (*p* < 0.05) between CANS and CVR indices of CCC. The “n” columns list the number of patients available for the given pairwise correlation analysis.

CVR Indices	VHRR	n	SI	n	PRT	n
	(Spearman’s Correlation Coefficient; *p*-Value)					
RTB_ICAop_	0.046; 0.876	14	0.307; 0.265	15	0.169; 0.564	14
THRR_PSVICAop_	−0.279; 0.277	17	0.121; 0.633	18	−0.028; 0.918	17
THRR_MBFVICAop_	−0.125; 0.633	17	−0.176; 0.484	18	0.092; 0.735	16

**Table 3:** Results of Spearman’s pairwise correlation test (*p* < 0.05) between CANS and CVR indices of HV-BH. Significant differences (*p* < 0.05) are indicated in **bold** numbers. The “n” columns list the number of patients available for the given pairwise correlation analysis.

CVR Indices	VHRR	n	SI	n	PRT	n
	(Spearman’s Correlation Coefficient; *p*-Value)					
HVI_ICAop_	−0.192; 0.404	21	0.045; 0.834	24	−0.285; 0.211	21
timetoMBFV_HVminICAop_	0.163; 0.481	21	−0.032; 0.881	24	−0.013; 0.954	21
BHI_ICAop_	0.081; 0.729	21	−0.369; 0.076	24	0.108; 0.640	21
CAR_timetomaxICAop_	**0.450; 0.041**	21	−0.027; 0.900	24	−0.243; 0.288	21
CAR_timetominICAop_	0.081; 0.729	**21**	**0.474; 0.019**	24	0.025; 0.913	21

**Table 4:** Results of Spearman’s pairwise correlation test (*p* < 0.05) between CANS and CVR indices of the VM. Significant differences (*p* < 0.05) are indicated in **bold** numbers. The “n” columns list the number of patients available for the given pairwise correlation analysis.

CVR Indices	VHRR	n	SI	n	PRT	n
	(Spearman’s Correlation Coefficient; *p*-Value)					
time to 2b_ICAop_	−0.109; 0.565	30	0.187; 0.297	33	0.144; 0.439	31
time to OS_ICAop_	−0.219; 0.244	30	0.026; 0.886	33	0.244; 0.185	31
CPOI_ICAop_	−0.008; 0.966	29	0.133; 0.467	32	0.340; 0.061	31
CPVR_ICAop_	0.046; 0.807	30	0.105; 0.562	33	−0.164; 0.379	31
CVAR_ICAop_	−0.172; 0.373	29	0.230; 0.205	32	**−0.529; 0.002**	31

**Table 5:** Canonical correlation coefficients.

Canonical Variates	Correlation	Test Statistic	*p*-Value
1–3	0.782	1.585	0.392
2–3	0.725	0.973	0.454
3	0.668	0.447	0.410

## Data Availability

The raw data supporting the conclusions of this article will be made available by the authors on request.

## Abbreviations

The following abbreviations are used in this manuscript:

ABP	Arterial Blood Pressure
BFV	Blood Flow Velocity
BH	Breath-holding test
BHI	Breath-holding Index
BRS	Baroreflex Sensitivity
CANS	Cardiovascular Autonomic Nervous System
CAR	Cerebral Arterial Resistance
CAR-AUC	Cerebral Arterial Resistance Area Under the Curve
CCA	Common Carotid Artery
CEA	Carotid Endarterectomy
CPOI	Centro-Peripheral Overshoot Index
CVAR	Cerebrovascular Valsalva Ratio
CVR	Cerebrovascular Reactivity
ECG	Electrocardiogram
HR	Heart Rate
HV	Hyperventilation
HVI	Hyperventilation Index
ICA	Internal Carotid Artery
ICAop	The To-Be Operated Side
ICAnonop	The Side Not To Be Operated On
ICAS	Internal Carotid Artery Stenosis
MBFV	Mean Blood Flow Velocity
MCA	Middle Cerebral Artery
NASCET	North American Symptomatic Carotid Endarterectomy Trial
OS	Overshoot
PRT	Pressure Recovery Time
RTB	Return to Baseline
SI	Symphatetic Index
TCD	Transcranial Doppler Sonography
THR	Transient Hyperaemic Response
THRR	Transient Hyperaemic Response Ratio
VHRR	Valsalva Heart Rate Ratio
VM	Valsalva Maneuver
